# Health-Related Behaviors and Perceived Health Status According to Water and Sugar-Sweetened Beverage Intake in Korean Adolescents

**DOI:** 10.3390/nu16173038

**Published:** 2024-09-09

**Authors:** Yoon Sun Kim, Hyun Ja Kim

**Affiliations:** 1 Department of Home Economics Education, College of Education, Kangwon National University, 1 Gangwondaehak-gil, Chuncheon-si 25913, Gangwon State, Republic of Korea; lava777@kangwon.ac.kr; 2 Department of Food and Nutrition, Gangneung-Wonju National University, 7 Jukheon-gil, Gangneung-si 25457, Gangwon State, Republic of Korea

**Keywords:** water intake, sugar-sweetened beverage, adolescents, health-related behaviors, perceived health status

## Abstract

The objective of this study was to determine the relationship between water and sugar-sweetened beverage (SSB) intake, health behaviors, and self-perceived health status using data from the 2019 Korea Youth Risk Behavior Web-based Survey (KYRBS). The subjects included in this analysis were 57,302 Korean adolescents from the 7th to 12th grades. The intake patterns of water and SSBs were categorized into four groups: Group I, adequate water intake (≥4 cups/day) and low frequency of SSB intake (≤1–2 times/week); Group II, adequate water intake and high frequency of SSB intake; Group III, inadequate water intake (<4 cups/day) and low frequency of SSB intake; Group IV, inadequate water intake and high frequency of SSB intake (≥3 times/week). Complex sample analyses were used for considering strata, clusters, and weights for samples. Significant differences were observed in the distribution of sociodemographic characteristics between the water and SSB intake groups. As grade levels increased or if students were female, there was a significant increase in the proportion of students characterized by low water intake and high consumption of SSB. Adolescents with healthier beverage habits, characterized by adequate water intake and low frequency of SSB consumption (Group I), generally abstained from smoking and alcohol, were more physically active, and maintained a desirable diet, reporting a better perceived health status. In contrast, those with higher SSB consumption and inadequate water intake (Group IV) were more likely to perceive their health as poor, with higher rates of smoking and alcohol use, lower physical activity levels, and poorer dietary habits compared to Group I. In conclusion, adolescents with desirable beverage consumption habits differed by sex and grade and they reported positive health behaviors and better overall health status. This suggests that there is a need for more active education and intervention in schools and families, as well as increased efforts by adolescents to promote healthy beverage habits.

## 1. Introduction

Adolescence is a critical period of rapid physical and mental growth that requires adequate nutrient intake. Establishing healthy eating habits during this time is important, as dietary behaviors formed during adolescence often persist into adulthood [1]. Compared to childhood, adolescence involves significant dietary transitions, including increased autonomy over food choices and a higher tendency toward irregular eating patterns. These transitions influence not only food consumption but also beverage choices [2]. The types of beverages consumed, such as water or sugar-sweetened beverages (SSBs), can be linked to overall health. Understanding the relationship between beverage choices and health during this formative period can provide evidence for promoting better beverage-drinking habits among adolescents.

In Korea, the nutrition-related health status and behaviors of adolescents are increasingly concerning. Analyses of the Korea Youth Risk Behavior Web-based Survey (KYRBS) reveal that many Korean adolescents fail to meet the recommended daily intake of fruits and vegetables [3] and often consume high-calorie and low-nutrient foods [4]. For these reasons, it has been suggested that the prevalence of overweight and obesity among Korean adolescents is rising [5]. This increase in obesity might be linked to excessive sugar intake from the high consumption of SSBs. These dietary behaviors underscore the need for interventions to improve nutritional status and promote healthier eating and beverage habits among Korean youth. 

Numerous beverages contain high sugar levels, and it has been reported that high beverage intake correlates with excessive sugar consumption, especially among teenagers [6,7]. Total sugar intake is highest among adolescents in the United States (12–19 years) [8,9], United Kingdom (11–18 years) [10], and Canada (14–18 years) [11], raising global concerns about adolescent sugar intake. This is problematic because SSBs contribute to obesity [12,13,14], cavities [15,16], and fractures [17], all of which can interfere with adolescents’ growth and development and negatively affect their diet quality, due to the lack of trace nutrients such as calcium [18,19].

Rosinger et al. (2019) suggested that daily water consumption is necessary to avoid excessive calorie and sugar intake [20]. Therefore, maintaining an adequate daily water intake is important for supporting healthy dietary habits and proper metabolic function. Previous studies [21,22,23,24] have primarily examined the relationship between either water or SSB intake and health-related behaviors, as well as dietary intake, without considering both together in terms of the overall beverage consumption patterns of adolescents. Therefore, research exploring the combined effects of water and SSB intake is necessary to provide a more comprehensive understanding of how, overall, beverages collectively influence adolescent health. 

Perceived health status, an indicator of an individual’s objective health, has been associated with smoking, alcohol consumption, regular physical activity, weight loss, and mental health. Understanding perceived health status is crucial as it reflects not only physical well-being but also mental and emotional health, influencing overall quality of life [25,26,27]. Previous studies [21,22,23] have shown that the intake of water or SSBs can be significantly related to physical health outcomes. However, few studies have analyzed the relationship between water and SSB intake and perceived health status after adjusting for these health-related behaviors. Also, in previous studies [21,22,28] using data from the KYRBS for SSBs, water intake was not considered. Exploring this relationship can enhance our understanding of how beverage consumption patterns are related to adolescents’ health, highlighting the importance of promoting healthy beverage habits.

Therefore, the objectives of this study were to determine whether water and SSB intake is related to sociodemographic characteristics, health-related behaviors such as smoking, alcohol consumption, physical activity, and dietary intake, and perceived health status in representative Korean adolescents, using data from the 2019 KYRBS, which, for the first time, included a question about water intake.

## 2. Materials and Methods

### 2.1. Study Population

The raw data [29] from the 2019 KYRBS were used for this secondary analysis of the cross-sectional study. The KYRBS was a national, self-reported online survey conducted during school hours with the cooperation of supervising teachers. It targeted middle (7th–9th-grade) and high school (10th–12th-grade) students in South Korea. In the 2019 survey, 57,303 (95.3% participation rate) out of 60,100 students from 800 sample schools across 17 cities and provinces participated. After excluding 1 person’s response, which had missing values for the water intake query, a total of 57,302 students were included in the final analysis. The KYRBS, a government-approved statistical survey, was approved by the Institutional Review Board (IRB) of the Korean Centers for Disease Prevention and Control up until 2014. Since 2015, the study has been conducted without IRB review, in accordance with the Enforcement Regulations of the Bioethics and Safety Act. Parental consent is not required for KYRBS response as no personal information is collected during the survey. Therefore, to perform this secondary data analysis study, IRB exemption approval was obtained from Kangwon University (KWNUIRB-2021-12-004). 

### 2.2. Questionnaires in the KYRBS

The KYRBS is an anonymous self-written online survey conducted every year to understand the health-related behavior status of Korean adolescents. Of the 15 domains of the 2019 KYRBS questionnaires, 6 domains were investigated in our study: sociodemographic characteristics, smoking, alcohol drinking, physical activity, dietary intake, and perceived health status. 

Water Intake: Water intake was measured using the following question: “How often have you drunk water every day in the past 7 days?” The original response scales were “<1 cup (200 mL)/day”, “1 to 2 cups/day”, “3 cups/day”, “4 cups/day”, and “≥5 cups/day”. Water intake was defined as ≥4 cups/day (adequate intake group) or <4 cups/day (inadequate intake group), based on the median water intake for adolescents in the 2013–2017 Korea National Health and Nutrition Examination Survey [5] and the results of water intake studies conducted on Korean adolescents [30].

SSB Intake: An SSB was defined as a sugar-added beverage, including soda, fruit drinks, coffee drinks, sweetened milk, sports drinks, and energy drinks [31]. Among the KYRBS questionnaire items, three items regarding high-caffeine drinks, energy drinks, and sweetened drinks were used to determine SSB intake: “How often have you had carbonated drinks in the past 7 days?”, “How often have you drunk energy (or high-caffeine) drinks in the past 7 days?”, and “How often have you had sugary beverages (excluding carbonated drinks and energy (high-caffeine) drinks) in the past 7 days?” The original response scale was “none”, “1–2 times/week”, “3–4 times/week”, “5–6 times/week”, “1 time/day”, “2 times/day”, and “≥3 times/day”. In our study, the weekly intake frequency of SSBs was divided into ≥3 and ≤1–2 times/week, with reference to a previous study [32]. 

The water intake and SSB intake results were combined and then classified into four groups: Group I, adequate water intake (≥4 cups/day) and low frequency of SSB intake (≤1–2 times/week); Group II, adequate water intake (≥4 cups/day) and high frequency of SSB intake (≥3 times/week); Group III, inadequate water intake (<4 cups/day) and low frequency of SSB intake (≤1–2 times/week); Group IV, inadequate water intake (< 4 cups/day) and high frequency of SSB intake (≥3 times/week).

Sociodemographic characteristics and BMI status: Sex, grade, parents’ educational level, economic status, residence type, subjective academic performance, and BMI were included in the questionnaire. The original response scale for parents’ educational level was “college graduate or higher”, “high school graduate”, “middle school graduate or lower”, and “not sure”. In our study, “college graduate or higher” was considered to indicate a higher education level. Economic status was reclassified from the original response scale of “high”, “upper middle”, “middle”, “lower middle”, and “low” into three categories: upper (combining “high” and “upper middle”), middle (“middle”), and lower (combining “lower middle” and “low”). Academic performance was measured with the question, “How would you describe your academic performance over the past 12 months?” Based on the original response scale of “high”, “upper middle”, “middle”, “lower middle”, and “low”, it was also reclassified into three categories: upper (combining “high” and “upper middle”), middle (“middle”), and lower (combining “lower middle” and “low”). The “upper” category was considered to indicate great academic performance. The body mass index (BMI), calculated using height and weight, was divided into underweight (<5th percentile), normal (5th–84th percentile), overweight (85th–94th percentile), and obese (≥95th percentile), based on the growth chart percentiles for children and adolescents in South Korea [33].

Health-related Behaviors: Smoking was assessed based on the item: “Have you ever smoked even a few puffs of a cigarette?” The responses were divided into “yes” or “no”. Alcohol drinking was assessed using the item: “Have you ever had at least one drink of alcohol?” The responses were divided into “yes” or “no”. Physical activity was assessed based on the question: “How many days have you had at least 60 min a day of physical activity with an increased heart rate or a shortness of breath in the past 7 days?” The original response scale was “none”, “1 day/week”, “2 days/week”, “3 days/week”, “4 days/week”, “5 days/week”, “6 days/week”, and “7 days/week”. In our study, physical activity for at least 60 min per day was classified into ≥5 and <5 days/week with reference to the Physical Activity Guidelines for Children and Adolescents in Korea [34]. 

Dietary Intake: Breakfast intake, fruit intake, vegetable intake, milk intake, fast food intake, and eating convenience foods were included. Breakfast intake was measured using the question: “How many days have you had breakfast in the past 7 days?” Breakfast intake frequency was divided into ≥5 and <5 days/week on the original response scale from 0 to 7 days/week. Fruit and vegetable intakes were assessed using the respective questions: “How often have you eaten fruits (excluding fruit juices) in the past 7 days?” and “How often have you eaten vegetables (excluding kimchi) during meals in the past 7 days?” Intake frequency of fruits and vegetables was divided into <3, 3–6, and ≥7 times/week [3]. Milk intake was assessed using the question: “How often have you had white milk or processed milk (including chocolate milk, coffee milk, banana milk, strawberry milk) in the past 7 days?” Intake frequency of milk or processed milk was divided into <7 and ≥7 times/week. Fast food and convenience food intakes were assessed using the respective questions: “How often have you eaten fast food in the past 7 days?” and “How many times have you eaten foods sold at a convenience store, supermarket, and school canteen (referred to as convenience foods) as your meals in the past 7 days?” Intake frequencies were divided into <3 and ≥3 times/week. The original response scale of fruit intake, vegetable intake, milk intake, fast food intake, and eating convenience foods was “none”, “1–2 times/week”, “3–4 times/week”, “5–6 times/week”, “1 time/day”, “2 times/day”, and “≥3 times/day”.

Perceived Health Status: Perceived health status has been reported to be in good agreement with objective health status [25,27], and, in our study, it was measured using one item: “In general, how would you rate your health status?” The respondents answered on a five-point scale of 1 (very good) to 5 (very poor). Perceived health was classified into three categories: good (1–2 points), average (3 points), and poor (4–5 points).

### 2.3. Statistical Analyses 

The KYRBS uses a complex sample design; therefore, complex sample analyses were performed using strata, clusters, and weights [29]. Statistical analysis was performed using SAS, version 9.4 (SAS Institute Inc., Cary, NC, USA). The differences in sociodemographics according to water and SSB intake groups were analyzed using the Rao–Scott χ^2^ test [35]. The health-related behaviors, dietary intake, and perceived health status according to the intake of water and SSBs were calculated using surveylogistic regression after adjusting for sex (male or female), grade (middle school 1 to high school 3), parents’ education level (≥college, ≤high school, or not sure/missing), economic status (upper, middle, or lower class), residence type (living with or not living with family), academic achievement (upper, middle, or lower class), BMI (underweight, normal, overweight, obesity, or missing), smoking (yes or no), alcohol drinking (yes or no), moderate physical activity (≥5 or <5 days/week), breakfast intake (≥5 or <5 days/week), fruit intake (<3, 3–6, or ≥7 times/week), vegetable intake (<3, 3–6, or ≥7 times/week), milk intake (<7 or ≥7 times/week), fast food intake (<3 or ≥3 times/week), and convenience food intake (<3 or ≥3 times/week). The odds ratio (OR) and 95% confidence interval (95% CI) of perceived poor health according to the intake of water and SSBs were calculated using a multivariable surveylogistic regression model after adjusting for sex, grade, economic status, parents’ education level, residence type, smoking, alcohol drinking, moderate physical activity, breakfast intake, fruit intake, vegetable intake, milk intake, fast food intake, and convenience food intake. All the analyses were two-tailed, and the significance level was set to *p* < 0.05.

## 3. Results

### 3.1. Sociodemographic Characteristics and BMI Status Related to Water and SSB Intake

Table 1 shows the sociodemographic characteristics of adolescent participants according to water and SSB intake. Group II, characterized by adequate water and high frequency of SSB intake, had a high proportion of males (65.9%), while Group III, characterized by inadequate water and low frequency of SSB intake, had a high proportion of females (65.4%). Group I, which was characterized by adequate water intake and low frequency of SSB intake, comprised largely middle school students (55.1%), whereas Group IV, with inadequate water and high frequency of SSB intake, had a high proportion of high school students (57.6%). Group I also had a high proportion of students whose parents had a high level of education (37.2% for fathers and 36.0% for mothers), higher economic status (42.9%), lived with their families (96.3%), and reported great academic performance (41.9%). In contrast, Group IV had a higher proportion of students with lower economic status (13.3%), who did not live with their families (5.3%), and had poor academic performance (34.2%). Group III had a high proportion of underweight (19.6%) and normal-weight (66.1%) students, while Group II had a high proportion of obese (8.9%) students.

### 3.2. Health-Related Behaviors According to Water and SSB Intake

Table 2 shows the students’ health-related behaviors according to water and SSB intake. Compared to Group I, Groups II and IV, who frequently drank SSBs regardless of water intake, had a higher proportion of smoking (7.7% for Group I vs. 13.2% for Group II and 11.1% for Group IV) and alcohol drinking (32.4% for Group I vs. 42.5% for Group II and 38.5% for Group IV) (*p* < 0.001). Groups I and II, who drank adequate water regardless of SSB intake, had a high proportion of respondents who exercised at least 60 min/day for ≥5 days/week (15.8% and 17.6%, respectively), compared to Group III and Group IV, who drank inadequate amounts of water (7.6% and 8.7%, respectively) (*p* < 0.001). 

### 3.3. Dietary Intake According to Water and SSB Intake

Table 3 shows the dietary intake according to water and SSB intake. Group I had a higher proportion of participants eating breakfast ≥ 5 days/week (56.2%), consuming fruits ≥ 7 times/week (21.8%), vegetables ≥ 7 times/week (45.3%), and milk ≥ 7 times/week (21.3%) compared to those in Group IV (44.4%, 17.5%, 30.2%, and 20.8%, respectively). Compared to Group I, Groups II and IV, who frequently consumed SSBs regardless of water intake, had a higher frequency of fast-food consumption (11.8% for Group I vs. 32.2% for Group II and 31.5% for Group IV) and convenience food consumption (14.7% for Group I vs. 34.7% for Group II and 36.6% for Group IV) (*p* < 0.001).

### 3.4. Perceived Health Status According to Water and SSB Intake

Table 4 shows the perceived health status according to water and SSB intake. Group I had the highest proportion of adolescents who reported their health status as “good” (74.1%), which was significantly higher than the proportion who reported this in Group IV (69.2%) (*p* < 0.001). Table 5 shows the risk of perceived poor health, according to water and SSB intake. Overall, the risk of perceived poor health was higher in Group IV (OR = 1.26, 95% CI = 1.14–1.38) compared to Group I. The significantly increased risk of perceived poor health in Group IV was observed regardless of sex, grade, and economic status. In more detail, the risk of perceived poor health was significantly higher in Group IV compared to Group I in both males (OR = 1.35, 95% CI = 1.16–1.57) and females (OR = 1.21, 95% CI = 1.07–1.38), as well as in both middle school students (OR = 1.28, 95% CI = 1.09–1.50) and high school students (OR = 1.23, 95% CI = 1.09–1.39). Moreover, when compared to Group I, the risk of perceived poor health in Group IV was higher in both the upper (OR = 1.27, 95% CI = 1.06–1.52) and the middle economic classes (OR = 1.28, 95% CI = 1.11–1.47), but was not significant in the lower economic classes (OR = 1.22, 95% CI = 0.97–1.53).

## 4. Discussion

Using raw data from the 2019 KYRBS, we observed significant differences in the distribution of sociodemographic characteristics across water and SSB intake groups. Furthermore, adolescents who displayed the most desirable beverage consumption behavior (adequate water intake and low frequency of SSB intake) also showed positive health behaviors, such as abstaining from smoking and alcohol, engaging in physical activity, and maintaining better dietary intake behaviors. They also reported a better perceived health status.

In our study, the group that consumed less water and fewer SSBs had a higher proportion of females, and the group that consumed more water and more SSBs had a higher proportion of males. This aligns with a study on water intake among adolescents aged 10–17 years across 13 countries. Despite variations in water intake levels based on age and sex across these nations, it was observed that, on average, female students consumed less water than their male counterparts [36]. Such disparities may arise from differences in metabolism and physical activity levels between the two sexes. Also, in several studies of adolescents, a higher intake of SSBs was found among male students compared to female students [20,21,23].

Similar to our study, desirable beverage consumption has been reported to be associated with lower grades, higher education levels in parents, higher economic status, living with family, and superior academic performance [8,9,10,11,23,24,37,38,39]. Past research has frequently emphasized the influence of socioeconomic factors as significant determinants of dietary and beverage choices among young people [9,11,23,24,38]. Additionally, these studies have found a strong correlation between family environment and an adolescent’s dietary habits. This suggests that positive family dynamics can play a protective role in promoting healthier food and beverage consumption.

In our study, the group with inadequate water intake, regardless of SSB intake, showed higher obesity rates compared to the group with adequate water intake, indicating a potential link between water intake and obesity. Although evidence on the impact of water intake on weight management in youth is limited, plain water could play a significant role in reducing energy intake and potentially preventing obesity [40].

In our study, regardless of water intake, the groups that frequently consumed SSBs were more likely to smoke and drink alcohol compared to the group with less frequent SSB consumption. This suggests an association between a high frequency of SSB intake and both smoking and alcohol drinking. Studies from the United States also showed a strong association between the frequency of SSB consumption and both alcohol drinking and smoking among adolescents [38,41]. It is more likely that adolescents who ignore the known health risks of smoking and alcohol consumption are also more likely to ignore the known health risks of consuming SSBs. These findings suggest that undesirable beverage consumption could compound the problems of smoking and alcohol consumption, further increasing health risks among adolescents. This association between SSB intake and smoking or alcohol consumption was observed in both male and female adolescents in our stratified analysis by sex.

In our study, regardless of SSB intake, groups who drank adequate amounts of water had a high rate of exercising at least 60 min/day for ≥5 days/week. In a study on high school students in the United States, adolescents who engaged in at least 60 min of physical activity a day for ≥5 days/week showed a higher rate of adequate water intake (≥4 cups/d) than those with lower physical activity frequency, indicating that the adequate intake of water is associated with regular exercise [42].

In our study, adolescents with desirable beverage consumption patterns were found to have better dietary intake behaviors, including regular breakfasts, daily fruits, and vegetables. Conversely, adolescents who frequently consumed SSB, especially regardless of water intake, were found to have a higher frequency of consuming fast food (≥3 times/week) and eating at convenience stores, supermarkets, or school canteens (≥3 times/week). A study using data from the National Health and Nutrition Examination Survey (NHANES) in the United States reported that adolescents (aged 12–18 years) who drank more than two servings of water daily (one serving being 8 oz) and those with lower SSB intake had better dietary quality than those who did not [24]. This finding suggests that good dietary quality might be associated with adequate water intake and lower SSB intake. In contrast, studies have shown a strong association between SSB intake and fast-food consumption among children and adolescents [43,44]. The combination of sugar and fat enhances the sensory appeal and palatability of foods, especially for children, who often prefer sweeter and higher-fat options. This synergy may explain the frequent pairing of SSBs with high-fat fast foods [45]. This could be related to the frequent co-advertising of SSBs with fatty foods. Additionally, this integrated marketing strategy, which often emphasizes cost-saving bundles, is prevalent not only in Korea but also globally.

In our study, adolescents with undesirable beverage consumption patterns were more likely to perceive their health as poor (OR = 1.26, 95% CI = 1.14–1.38). This suggests an association between beverage consumption and health status. Although comprehensive investigations into this relationship, especially among adolescents, are somewhat sparse, both domestically and abroad, there are some indications from prior research. Some studies focusing on older demographics, like college students and adults, have hinted that regular soft drink consumption might be associated with a more negative self-assessment of health [46]. In contrast, it is believed that the perception of health risks might discourage individuals from excessive SSB consumption [47], although more studies are warranted to affirm this. Our study found that both inadequate water intake and frequent SSB intake were significantly associated with a higher risk of perceived poor health. These risks were significant regardless of sex or grade. Additionally, the risk of perceived poor health was significantly increased in Group 4, with less desirable beverage consumption in the upper (OR = 1.27, 95% CI = 1.06–1.52) and middle economic classes (OR = 1.28, 95% CI = 1.11–1.47). However, this association was not significant in the lower economic classes (OR = 1.22, 95% CI = 0.97–1.53), possibly because other factors influencing health had a greater impact than beverage consumption. In fact, when comparing Model 2, adjusting for various risk factors, with Model 1, adjusting for only sex and grade in Table 5, notable drops in the OR values were observed. This suggests that other health-related risk factors, such as sociodemographic factors and behavioral factors, might significantly affect the risk of perceived poor health. In particular, the lower economic classes may have more concentrated risk factors, so the impact of SSBs on perceived health may be relatively less. In examining the relationship between sociodemographic factors and health-related behaviors based on economic status, it was found that in the lower economic classes, parents’ education level (*p* < 0.001) was lower, and the proportion of smoking (*p* < 0.001) and alcohol drinking (*p* < 0.001) was higher. These specific associations warrant further exploration. While not directly related but still of relevance, a longitudinal study spanning 15 years noted a potential consequence of these health perceptions [48]. It suggested that individuals with negative health perceptions during their younger years might be predisposed to health complications, including chronic conditions, as they age. This underscores the importance of understanding and potentially rectifying these perceptions early on. 

Given the association we found between higher parental education levels and better beverage intake habits, providing dietary guidance and knowledge to parents could enhance adolescents’ beverage choices and thereby improve their health perception. Particularly, the consumption of SSBs was observed to increase more among high school students compared to middle school students. In Korean high schools, where the emphasis is heavily on academics, proper nutritional education is lacking. Hence, there is a greater need for comprehensive health behavior education, including the topic of water and beverage consumption, specifically targeted at high school students.

Our study had a few limitations. First, it was a cross-sectional study; thus, we could determine only associations, not causations. Second, processed milk, despite being defined as a sweetened drink, was excluded from the sweetened drink category. This was because it was already accounted for in the question regarding the frequency of milk intake in the raw data. Third, the raw data contained only an item on SSB intake frequency; therefore, we could not analyze the amount of SSB intake. Fourth, among the various survey questions on alcohol and smoking, this study chose to analyze lifetime consumption experience, not allowing for differentiation between varying frequencies and amounts of use, and not reflecting current behaviors. Fifth, physical activity was evaluated with a single question based on recommendations rather than a validated tool for this population. Finally, the study assessed intake frequency but not the amounts consumed, so participants who reported less frequent consumption might have consumed larger or equal amounts compared to those who reported more frequent consumption. Future research should include comprehensive questions on sweetened beverages, measure both the frequency and quantity of SSB intake, use validated tools for evaluating physical activity, and reflect current behaviors in evaluations of alcohol and smoking. Despite these limitations, this study has significant strengths. It presents the results of a large-scale national survey; thus, the findings can serve as reliable evidence of the factors related to water and SSB intake in Korean adolescents.

## 5. Conclusions

Desirable beverage consumption habits may be related to parental education levels or economic status, and were aligned with other positive health behaviors, such as refraining from smoking and alcohol, participating in physical activity, and maintaining a healthier diet. Students with these habits also reported better overall health status. Therefore, more active education and intervention in schools and families are needed, along with increased efforts by adolescents to promote healthy beverage habits.

## Figures and Tables

**Table 1:** Sociodemographic characteristics and BMI statuses of adolescent participants, shown according to water and sugar-sweetened beverage intake groups.

	Total (*n* = 57,302)	Categories of Water and Sugar-Sweetened Beverage Intake ^1^				*p*-Value ^4^
		I (*n* = 10,572) ^2^	II (*n* = 19,963)	III (*n* = 8380)	IV (*n* = 18,387)	
	% (SE) ^3^	% (SE)	% (SE)	% (SE)	% (SE)	
Sex						<0.001
Male	52.0(1.2)	52.7(1.4)	65.9(1.2)	34.6(1.4)	44.3(1.4)	
Female	48.0(1.2)	47.3(1.4)	34.1(1.2)	65.4(1.4)	55.7(1.4)	
Grade						<0.001
Middle school (7th)	15.9(0.3)	21.4(0.6)	16.4(0.4)	16.7(0.5)	12.1(0.3)	
Middle school (8th)	15.3(0.3)	16.9(0.5)	16.1(0.4)	14.8(0.5)	13.9(0.4)	
Middle school (9th)	16.6(0.3)	16.8(0.4)	16.7(0.4)	16.5(0.6)	16.4(0.4)	
High school (10th)	17.1(0.3)	14.6(0.5)	16.6(0.4)	17.4(0.6)	19.0(0.4)	
High school (11th)	16.5(0.3)	14.1(0.5)	15.8(0.4)	16.7(0.6)	18.4(0.4)	
High school (12th)	18.5(0.3)	16.2(0.5)	18.4(0.4)	18.0(0.6)	20.2(0.4)	
Father’s educational level						<0.001
College or higher	35.9(0.4)	37.2(0.7)	34.8(0.6)	38.5(0.7)	35.3(0.6)	
High school or below	15.8(0.3)	15.2(0.5)	16.5(0.4)	15.5(0.5)	15.6(0.4)	
Not sure	12.0(0.2)	13.2(0.4)	11.3(0.3)	12.6(0.4)	11.9(0.3)	
Missing	36.2(0.4)	34.4(0.7)	37.4(0.5)	33.4(0.7)	37.2(0.6)	
Mother’s educational level						<0.001
College or higher	34.4(0.4)	36.0(0.7)	32.9(0.5)	37.4(0.7)	33.9(0.6)	
High school or below	18.6(0.3)	17.7(0.5)	18.7(0.4)	18.9(0.6)	18.6(0.4)	
Not sure	11.1(0.2)	12.1(0.4)	11.1(0.3)	10.9(0.3)	10.7(0.3)	
Missing	35.9(0.4)	34.2(0.7)	37.3(0.5)	32.9(0.7)	36.8(0.6)	
Economic status						<0.001
Upper class	39.7(0.4)	42.9(0.6)	42.1(0.5)	36.6(0.7)	36.7(0.5)	
Middle class	47.8(0.3)	46.0(0.6)	45.6(0.5)	50.2(0.7)	50.0(0.4)	
Lower class	12.5(0.2)	11.1(0.3)	12.3(0.3)	13.2(0.5)	13.3(0.3)	
Residence type						<0.001
Living with family	95.4(0.3)	96.3(0.3)	95.8(0.3)	95.2(0.5)	94.7(0.4)	
Not living with family	4.6(0.3)	3.7(0.3)	4.2(0.3)	4.8(0.5)	5.3(0.4)	
Academic achievement						<0.001
Upper	38.1(0.3)	41.9(0.6)	38.0(0.4)	38.1(0.6)	36.0(0.4)	
Middle	30.1(0.2)	29.9(0.5)	29.9(0.4)	31.7(0.6)	29.9(0.4)	
Lower	31.8(0.3)	28.2(0.5)	32.2(0.4)	30.1(0.6)	34.2(0.4)	
BMI ^5^						<0.001
Underweight	16.3(0.2)	14.5(0.4)	13.7(0.3)	19.6(0.5)	18.5(0.3)	
Normal	64.5(0.2)	63.0(0.5)	63.2(0.4)	66.1(0.6)	66.0(0.4)	
Overweight	9.6(0.1)	11.6(0.3)	11.1(0.2)	7.9(0.3)	7.7(0.2)	
Obese	7.0(0.1)	8.2(0.3)	8.9(0.2)	4.7(0.2)	5.3(0.2)	
Missing	2.6(0.1)	2.8(0.2)	3.1(0.1)	1.6(0.1)	2.5(0.1)	

^1^ I: Adequate intake of water (≥4 cups/d) and low intake of sugar-sweetened beverages (SSBs) (≤1–2 times/wk); II: adequate intake of water and high intake of SSBs; III: inadequate intake of water (<4 cups/d) and low intake of SSBs; IV: inadequate intake of water and high intake of SSBs (≥3 times/wk); ^2^ unweighted sample size; ^3^ weighted percentage (SE): because of rounding, weighted percentages may not add up to 100%; ^4^ *p*-values were calculated by the Rao–Scott chi-square test; ^5^ weight status was defined as follows: underweight: <5th percentile, normal: 5–84th percentile, overweight: 85–94th percentile, obese: ≥95th percentile.

**Table 2:** Health-related behavior, shown according to water and sugar-sweetened beverage intake groups in adolescents.

	Total (*n* = 57,302)	Categories of Water and Sugar-Sweetened Beverage Intake ^1^				*p*-Value ^4^
		I (*n* = 10,572) ^2^	II (*n* = 19,963)	III (*n* = 8380)	IV (*n* = 18,387)	
	% (SE) ^3^	% (SE)	% (SE)	% (SE)	% (SE)	
Smoking						<0.001
Yes	13.8(0.3)	7.7(0.3)	13.2(0.3)	6.3(0.3)	11.1(0.3)	
No	86.2(0.3)	92.3(0.3)	86.8(0.3)	93.7(0.3)	88.9(0.3)	
Alcohol drinking						<0.001
Yes	39.4(0.4)	32.4(0.6)	42.5(0.5)	28.0(0.6)	38.5(0.5)	
No	60.6(0.4)	67.6(0.6)	57.5(0.5)	72.0(0.6)	61.5(0.5)	
Moderate physical activity						<0.001
<5 days/week	85.3(0.3)	84.2(0.5)	82.4(0.4)	92.4(0.3)	91.3(0.3)	
≥5 days/week	14.7(0.3)	15.8(0.5)	17.6(0.4)	7.6(0.3)	8.7(0.3)	

^1^ I: Adequate intake of water (≥4 cups/d) and low intake of sugar-sweetened beverages (SSBs) (≤1–2 times/wk); II: adequate intake of water and high intake of SSBs; III: inadequate intake of water (<4 cups/d) and low intake of SSBs; IV: inadequate intake of water and high intake of SSBs (≥3 times/wk); ^2^ unweighted sample size; ^3^ weighted percentage (SE): because of rounding, weighted percentages may not add up to 100%. ^4^ Adjusting for sex, grade, parents’ education level, economic status, residence type, academic achievement, and BMI, the significant differences in percentage between 4 groups were calculated using surveylogistic regression.

**Table 3:** Dietary intake, shown according to water and sugar-sweetened beverage intake groups in adolescents.

	Total (*n* = 57,302)	Categories of Water and Sugar-Sweetened Beverages Intake ^1^				*p*-Value ^4^
		I (*n* = 10,572)^2^	II (*n* = 19,963)	III (*n* = 8380)	IV (*n* = 18,387)	
	% (SE) ^3^	% (SE)	% (SE)	% (SE)	% (SE)	
Breakfast intake						<0.001
<5 days/week	50.4(0.3)	43.8(0.6)	50.0(0.4)	47.9(0.7)	55.6(0.4)	
≥5 days/week	49.6(0.3)	56.2(0.6)	50.0(0.4)	52.1(0.7)	44.4(0.4)	
Fruit intake						<0.001
<3 times/week	41.2(0.3)	39.3(0.5)	37.2(0.4)	45.3(0.7)	43.0(0.5)	
3–6 times/week	38.3(0.2)	37.3(0.5)	39.8(0.4)	35.9(0.6)	38.2(0.4)	
≥7 times/week	20.5(0.2)	21.8(0.5)	21.5(0.3)	17.6(0.5)	17.5(0.3)	
Vegetable intake						<0.001
<3 times/week	21.4(0.2)	16.4(0.4)	17.0(0.3)	24.5(0.5)	26.1(0.4)	
3–6 times/week	41.0(0.2)	37.7(0.6)	40.4(0.4)	41.7(0.6)	43.0(0.4)	
≥7 times/week	37.6(0.2)	45.3(0.6)	42.0(0.4)	33.1(0.6)	30.2(0.4)	
Milk intake						<0.001
<7 times/week	77.2(0.3)	78.7(0.5)	76.2(0.4)	78.3(0.5)	79.2(0.4)	
≥7 times/week	22.8(0.3)	21.3(0.5)	23.8(0.4)	21.7(0.5)	20.8(0.4)	
Fast food intake						<0.001
<3 times/week	74.5(0.2)	88.2(0.4)	67.8(0.4)	88.0(0.4)	68.5(0.4)	
≥3 times/week	25.5(0.2)	11.8(0.4)	32.2(0.4)	12.0(0.4)	31.5(0.4)	
Eating at a convenience store, supermarket, or school canteen						<0.001
<3 times/week	70.7(0.3)	85.4(0.4)	65.3(0.4)	83.9(0.5)	63.5(0.4)	
≥3 times/week	29.3(0.3)	14.7(0.4)	34.7(0.4)	16.1(0.5)	36.6(0.4)	

^1^ I: Adequate intake of water (≥4 cups/d) and low intake of sugar-sweetened beverages (SSBs) (≤1–2 times/wk); II: adequate intake of water and high intake of SSBs; III: inadequate intake of water (<4 cups/d) and low intake of SSBs; IV: inadequate intake of water and high intake of SSBs (≥3 times/wk); ^2^ unweighted sample size; ^3^ weighted percentage (SE): because of rounding, weighted percentages may not add up to 100%. ^4^ Adjusting for sex, grade, parents’ education level, economic status, residence type, academic achievement, and BMI, the significant differences in percentage between 4 groups were calculated using surveylogistic regression.

**Table 4:** Perceived health status, shown according to water and sugar-sweetened beverage intake groups in adolescents.

	Total (*n* = 57,302)	Categories of Water and Sugar-Sweetened Beverage Intake ^1^				*p*-Value ^4^
		I (*n* = 10,572) ^2^	II (*n* = 19,963)	III (*n* = 8380)	IV (*n* = 18,387)	
	% (SE) ^3^	% (SE)	% (SE)	% (SE)	% (SE)	
Perceived health						<0.001
Good	70.0(0.3)	74.1(0.5)	73.2(0.4)	71.5(0.6)	69.2(0.4)	
Average	22.6(0.2)	19.7(0.5)	20.2(0.3)	21.7(0.5)	23.2(0.4)	
Poor	7.5(0.1)	5.5(0.2)	5.8(0.2)	5.9(0.3)	6.5(0.2)	

^1^ I: Adequate intake of water (≥4 cups/d) and low intake of sugar-sweetened beverages (SSBs) (≤1–2 times/wk); II: adequate intake of water and high intake of SSBs; III: inadequate intake of water (<4 cups/d) and low intake of SSBs; IV: inadequate intake of water and high intake of SSBs (≥3 times/wk); ^2^ unweighted sample size; ^3^ weighted percentage (SE): because of rounding, weighted percentages may not add up to 100%. ^4^ Adjusting for sex, grade, parents’ education level, economic status, residence type, academic achievement, BMI, smoking, alcohol drinking, moderate physical activity, breakfast intake, fruit intake, vegetable intake, milk intake, fast food intake, and convenience food intake, the significant differences in percentage between 4 groups were calculated using surveylogistic regression.

**Table 5:** Risk of perceived poor health according to the intake of water and sugar-sweetened beverages in adolescents.

OR (95% CI) ^1^									
		Model 1 ^2^				Model 2 ^3^			
		Categories of Water and Sugar-Sweetened Beverage Intake ^4^				Categories of Water and Sugar-Sweetened Beverage Intake			
		I (*n* = 10,572)	II (*n* = 19,963)	III (*n* = 8380)	IV (*n* = 18,387)	I (*n* = 10,572)	II (*n* = 19,963)	III (*n* = 8380)	IV (*n* = 18,387)
Total subjects		1.00 (reference)	1.25(1.12–1.39) ***	1.19(1.05–1.34) **	1.61(1.45–1.79) ***	1.00 (reference)	1.07(0.97–1.18)	1.07(0.96–1.20)	1.26(1.14–1.38) ***
Sex	Male	1.00 (reference)	1.18(1.01–1.38) *	1.22(0.98–1.51)	1.67(1.42–1.97) ***	1.00 (reference)	1.07(0.92–1.24)	1.08(0.88–1.32)	1.35(1.16–1.57) ***
	Female	1.00 (reference)	1.34(1.15–1.55) ***	1.18(1.02–1.37) *	1.58(1.38–1.80) ***	1.00 (reference)	1.10(0.96–1.26)	1.08(0.94–1.24)	1.21(1.07–1.38) **
Grade	Middle school	1.00 (reference)	1.21(1.04–1.41) *	1.15(0.96–1.37)	1.68(1.43–1.98) ***	1.00 (reference)	1.01(0.87–1.17)	1.06(0.89–1.26)	1.28(1.09–1.50) *
	High school	1.00 (reference)	1.27(1.10–1.47) **	1.21(1.03–1.42) *	1.58(1.38–1.80) ***	1.00 (reference)	1.09(0.96–1.25)	1.07(0.93–1.24)	1.23(1.09–1.39) **
Economic status	Upper class	1.00 (reference)	1.27(1.05–1.53) **	1.22(0.98–1.51)	1.63(1.36–1.97) ***	1.00 (reference)	1.05(0.88–1.25)	1.13(0.92–1.38)	1.27(1.06–1.52) *
	Middle class	1.00 (reference)	1.21(1.03–1.42) *	1.11(0.93–1.33)	1.59(1.36–1.84) ***	1.00 (reference)	1.06(0.91–1.23)	1.04(0.89–1.23)	1.28(1.11–1.47) **
	Lower class	1.00 (reference)	1.25(0.98–1.58)	1.17(0.89–1.52)	1.53(1.20–1.96) **	1.00 (reference)	1.15(0.92–1.44)	1.06(0.83–1.36)	1.22(0.97–1.53)

^1^ Abbreviation: OR, odds ratio; 95% CI, 95% confidence interval; ^2^ Adjusting only for sex and grade. In the stratified analysis by sex, grade, or economic status, each of these variables was respectively excluded from the multivariable surveylogistic regression model. ^3^ Adjusting for sex, grade, economic status, parents’ education level, residence type, BMI, breakfast intake, fruit intake, vegetable intake, milk intake, fast food intake, convenience food intake, moderate physical activity, alcohol drinking, and smoking. In the stratified analysis by sex, grade, or economic status, each of these variables was respectively excluded from the multivariable surveylogistic regression model. ^4^ I: Adequate intake of water (≥4 cups/d) and low intake of sugar-sweetened beverages (SSBs) (≤1–2 times/wk); II: adequate intake of water and high intake of SSBs; III: inadequate intake of water (<4 cups/d) and low intake of SSBs; IV: inadequate intake of water and high intake of SSBs (≥3 times/wk). * p < 0.05, ** p < 0.01, *** p < 0.001.

## Data Availability

Releasing of the data by researchers is not legally permitted. All data can be found in the Korea Center for Disease Control and Prevention database. The Korea Center for Disease Control and Prevention allows any researcher who promises to comply with research ethics to access data. The data of this article can be downloaded from the website (https://www.kdca.go.kr/yhs/) (accessed on 15 January 2022) after agreeing to comply with research ethics.
